# Psychogenic Polydipsia Complicated to Hyponatremia Induced Seizure in Schizophrenia: A Case Report from Nepal

**DOI:** 10.1155/2019/6021316

**Published:** 2019-11-16

**Authors:** Pawan Sharma, Bigya Shah, Modnath Sangroula, Richan Jirel

**Affiliations:** ^1^Department of Psychiatry, Patan Academy of Health Sciences, School of Medicine, Lalitur, Nepal; ^2^Medical Officer, Arogin Health Care and Research Centre, Kathmandu, Nepal

## Abstract

Psychogenic polydipsia is one of the common cooccurrences with Schizophrenia and if not addressed can lead to fatal consequences. There are some evidences for pharmacological management of this condition but nonpharmacological management starting from psycho-education to behavioural modification therapy involving family members can be a very effective strategy. We report a case from Nepal where psychogenic polydipsia was complicated to hyponatremia and lead to seizure episodes. We emphasize on asking a routine question about polydipsia in every patient of Schizophrenia in clinics.

## 1. Introduction

Polydipsia conventionally defined as the excessive intake of liquids more than 3 liters per day is poorly understood and underdiagnosed among chronic psychiatric patients [1]. The researchers have identified polydipsia to be associated with schizophrenia as early as 1923 [2]. It is estimated that about 20% of patients with schizophrenia exhibit primary polydipsia and about 20% of these patients experience life-threatening hyponatremia [1]. Retrospective review of records has shown self-induced water intoxication as a cause of death in considerable number of patients with schizophrenia [3]. The mechanism of altered water metabolism is poorly understood in these cases [4]. Evidence show that the prevalence of water intoxication among polydipsic patients of Asian origin with schizophrenia to be low compared to Western studies [5]. We report a case of schizophrenia who presented to our tertiary hospital setting with polydipsia, hyponatremia and seizure episodes and was managed conservatively.

## 2. Case Presentation

A 39 years old male presented to the emergency department with a history of two episodes of seizure with altered consciousness. He was admitted in ICU with supportive management and relevant investigations were done. Detailed history taken from his father revealed he had a chronic psychiatric illness for last 20 years characterized by aggressive behaviour, muttering to self, fearfulness and decreased sleep. He also had disorganized behaviour in the form of not bathing and washing, drinking dirty water stored for the purpose of toilet use and collecting garbage without apparent reason. He had marked apathy and blunting of emotional responses that resulted in social withdrawal and lowering of social performance. He also had a history of irrelevant talk when trying to hold a conversation. Considering these symptoms, a DSM 5 diagnosis of Schizophrenia was made. Treatment history revealed he was maintaining well on tablet Risperidone 2 mg/day for last three years.

Apart from this, his father also gave a history of increased intake of water for last two years. He would drink around 5-6 liters of water per day that had increased to around 9-10 liters per day in the last few weeks. When asked, he wouldn't justify the amount of water intake but simply answered that he was thirsty and had an urge to drink water. He also had polyuria. On the day of presentation in our tertiary care center, he had two episodes of generalized tonic-clonic seizure. His investigation revealed values as shown in Table 1.

As per the laboratory reports he appears to meet the criteria for Syndrome of Inappropriate Antidiuretic Hormone Secretion (SIADH) [6]. However considering the presence of polyuria and no other secondary causes, a provisional diagnosis of psychogenic polydipsia with hyponatremia induced seizure was made and the differential of SIADH was considered. Hyponatremia was corrected over two days. Restriction of water intake was done at the hospital. He was continued on the same dose of Risperidone. Family members and patient were psycho-educated about effects of increased water intake and the need to restrict the same. Behavioral therapy for compulsive water drinking was started with the patient. The father was given a role of a co-therapist. The patient was advised to keep a log diary of intake and output of water. He was advised to decrease his water intake by observing the log. After discharge in a span of two months, two follow-up visits were made. The patient showed remarkable improvement in his water drinking, it reduced from 10 liters approx. to 5 liters/day as seen by input/output charting made by the patient and supervised by his father. The patient was maintaining well in one year follow up.

## 3. Discussion

Psychogenic polydipsia though commonly found with patients of schizophrenia can be a potentially fatal condition. There have been reports of life threatening conditions such as seizure, rhabdomyolysis, aspiration pneumonia, and crural compartment syndrome associated with psychogenic polydipsia [7–9]. There are also reports of death secondary to self-induced water intoxication in patients with schizophrenia [10]. The present case is an example which shows seizure as a complication of hyponatremia secondary to water intoxication caused by psychogenic polydipsia in a patient with diagnosed schizophrenia. It is a challenge to diagnose and manage the condition as patients themselves do not reveal and deny [11]. There have been various methods described in literature to treat psychogenic polydipsia. Use of medications such as demeclocycline, propranolol, captopril, and naloxone has shown inconsistent results [12]. Similarly, atypical antipsychotics such as risperidone and quetiapine have mixed results [11–13]. There is fair amount of evidence suggesting that clozapine is effective in reducing water intake [14], and that vasopressin type 2 antagonists which are primarily used to treat heart failure are very effective in rapidly reversing and preventing further cases of water intoxication [15].

Apart from pharmacological treatment, the behavioural modification has been widely used and consists of the water restriction program [11, 12]. Measures such as restriction of water intake with the help of family member and daily monitoring of the input, output, and electrolytes corrected this condition in our case. Such simple and inexpensive management by psycho-educating and involving family members as co-therapist is beneficial in lower resource settings where there are limited staffs. Furthermore, psychogenic polydipsia, like schizophrenia, has a relapsing course [12].Therefore, nonpharmacological measures and using family members into the treatment may have potential benefits for the long-term management. Our case imparts need to routinely assess water intake in patients with chronic illness such as schizophrenia so as not to miss psychogenic polydipsia. Since this has life threatening consequences, the case also highlights importance to its prompt diagnosis and management with water restriction involving family members. This has been among the very few reported cases from Nepal.

## Figures and Tables

**Table 1:** Summary of investigations done.

Investigations	Results	References
Complete hemogram	WNL	-
Serum Na^+^	100 mEq/L	135−145 mEq/L
Serum K^+^	3.9 mEq/L	3.5−5.1 mEq/L
Serum urea	24 mg/dL	15−45 mg/dL
Serum creatinine	1.0 mg/dL	0.8−1.3 mg/dL
Urine osmolality	109.2 mmol/kg	300−900 mmol/kg
Serum osmolality	145 mmol/kg	275−295 mmol/kg
Urine Na^+^	46 mmol/L	<20 mmol/L
Thyroid function test	WNL	
Blood sugar		
Fasting	72 mg/dL	<100 mg/dL
Post prandial	120 mg/dL	<140 mg/dL
Serum Cortisol	29.46 mcg/dL	43−22.4 mcg/dL
Ultrasonography whole abdomen	WNL	-
Echocardiography	WNL	-
CT scan head	WNL	-
MRI brain	WNL	-

WNL: Within Normal Limits.
